# Anouchka Grose: Apocalypse now

**DOI:** 10.1192/bjb.2020.46

**Published:** 2020-04-30

**Authors:** Claire Mckenna

**Figure:**
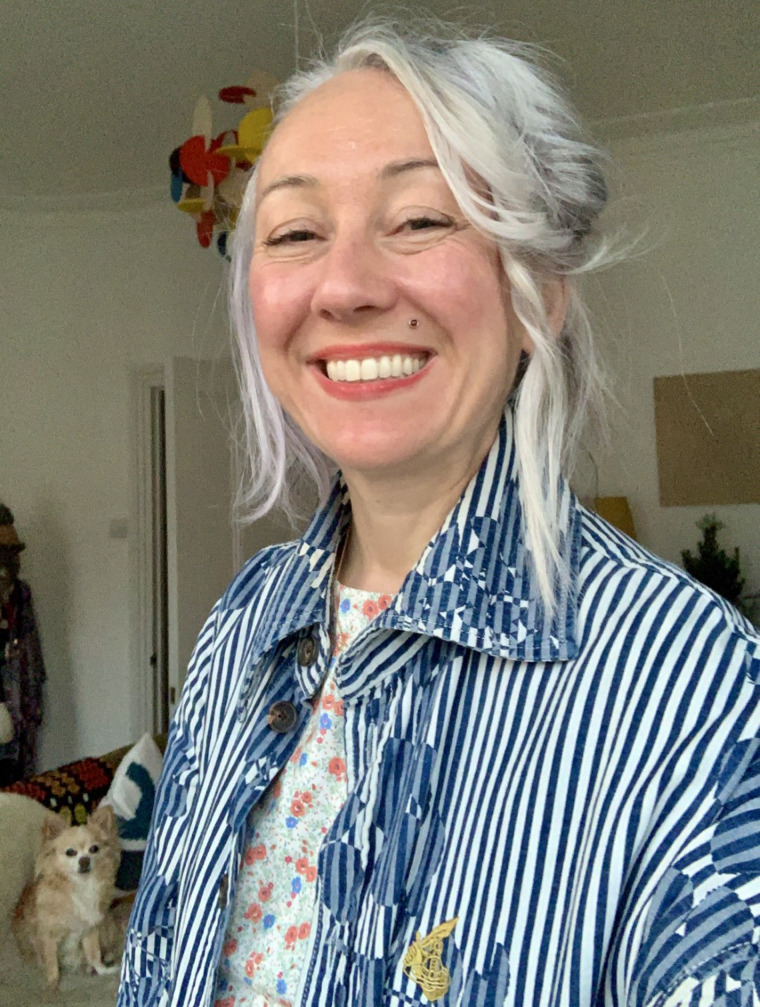

The apocalypse is trending. Climate catastrophe looms, end-times movie scenes spool out in real life all around us, and like a portent of our worst fears the COVID-19 pandemic is now upon us. As the scions of Big Bog Roll count their lucre, I've one pressing question for psychoanalyst Anouchka Grose – why toilet paper?! ‘It's like the last thing between us and barbarism’ she laughs, ‘once that's gone and you have to touch your own shit, it's like the whole of culture's collapsed then’!

Grose is refreshingly unguarded and swears liberally (a sign of her honesty and intelligence I think, as well as her Antipodean roots). She came to London from Sydney in the 1970s, aged 2, to live in ‘an Australian ghetto’ as she puts it. Her new book, *A Guide to Eco-Anxiety: How to Protect the Planet and Your Mental Health*, is to be published in May 2020, and she does worry a bit about how her irreverent tone will be received.^1^ It has tongue-in-cheek chapter titles like ‘Anxiety: freaking the fuck out’ and ‘Pleasure yourself: you know you want to’. She wrote it like that partly so the act of writing wasn't unbearable, ‘There's the question of how do you talk about something that's intolerable? And the climate thing *is* intolerable’. I think she's struck the balance in tone, which mitigates the nagging sense of fear.

Grose describes herself as a Lacanian psychoanalyst, who also lectures on Freud. She writes novels, non-fiction books, and journalism. Her new book originated from a piece she wrote in *The Guardian* in 2019, on the ecological crisis making Greenlanders depressed. However, she has long been an eco-warrior. She's been *mostly* vegetarian since 1985 and is a supporter of XR (Extinction Rebellion), the environmental movement that encourages ‘non-violent civil disobedience’.

The movie *Apocalypse Now* is based on the novel *Heart of Darkness*, Joseph Conrad's racist fever dream. His tale of rapacious Belgian colonialism in the Congo seems a fitting allegory for Grose's left-leaning world view. Like many, she links our rapacious consumerist culture both to the climate and COVID-19 crises, ‘Everyone seems as if they've seen behind the scenes of capitalism suddenly, like this window's open and you can see that it's just a stage’.

## Thoughts on humans and other animals

The parallels between climate-change anxiety and that related to COVID-19 have triggered a glut of hot-takes. I discuss with Grose that these crises induce an existential kind of dread. In the language we use about them, we hear the echo of primitive fears. Take the phrase ‘herd immunity’, for example. Grose says, ‘The word [herd] was shocking because it was dehumanizing. I mean, the idea that we're just a herd and some of us will be lost and that's fine. Like watching a nature documentary’.

In the language of scientists, however, she thinks there is a disconnect between their cautious approach and epoch-ending events like the climate crisis. She'd like them to be a little *more* alarmist, ‘I suppose they're trained not to, because of the idea of the impartiality of science, but it turns out that science is political’.

She lauds the approach of people like Professor Jem Bendell, a sustainability academic. His viral essay^2^ on climate-induced societal collapse didn't pull any punches. He writes, ‘With the power down, soon you wouldn't have water coming out of your tap…You will become malnourished… You will fear being violently killed before starving to death’.

## The struggle is real

I'm Skyping Grose at her home in London on a beautiful, clear, sunny Spring morning. I can hear birds chattering and see daffodils in bloom from my window beside the river in Belfast. It's hard to believe there's a deadly virus out there.

Grose recounts the story of a fellow analyst, who continues to see patients face to face (despite social distancing advice) because ‘he didn't want to get sucked into the hysteria’. Philosophical abstraction, Grose admits, can be unhelpful in dealing with concrete problems. This sometimes makes it difficult not to treat *all* prophets of doom as Cassandras, she thinks.

Do we have a duty as clinicians and therapists to protest the climate emergency? She thinks we do, ‘You know, if you're going to have subtle arguments, you need to have a world in which to have them. And so protecting that world, you know, it is absolutely primary’.

No man is an island, and this is where the COVID-19 crisis diverges sharply from the environmental one. XR, for example, is conspicuously a mass enterprise. ‘That's been one of the big things with the climate movement, that we have to collectivize’ she says, ‘we love each other, we have to be there for each other, touch each other. That's one of the pillars of the advice - to be communal. And suddenly that's been really, really problematized’.

The social isolation and poverty induced by the COVID-19 ‘lockdown’ may not bode well for our mental well-being. However, for some of Grose's patients, their anxiety has gone right down:

‘They don't have to make choices. They don't have to think they need to have a better job, look for a boyfriend, you know, all these sort of horrible choice making things have gone. …They're just thinking, well, whatever it is, you know at least it's interesting.’

## No pills for the pain

In her book on eco-anxiety, Grose says fear is a rational response to environmental Armageddon. ‘It's the people who aren't worried who are crazy’ she writes. The trick, she says, is to manage our anxiety so that we're on the helpful side of the Yerkes–Dodson stress curve. Her take-home message is to ‘…be anxious, be very anxious, because your anxiety can be a brilliant resource’.

She recognizes the paralysis that often comes with eco-anxiety, as we feel stuck between a compulsion to act and the futility of our individual efforts. She suggests a piecemeal approach to tackling climate change and also gives us permission to breathe. ‘It's vital to accept that there isn't a correct level of activity with regard to the climate’ she writes, ‘each person has to gauge what they can bear’.

In her book, she blends practical tips on environmentalism and personal anecdote, with strategies to soothe mind and body. As you would expect, she has some difficulty with cognitive–behavioural therapy framing real-life problems as something you can think yourself out of. About psychoanalysis, she says:

‘It's not that I think psychoanalysis has brilliant answers to everything. But I suppose the anti-normative thing and the idea that a cure doesn't have to be a quick fix, that you have these very open-ended speculative treatments, seems to me quite a good way to approach this stuff [eco-anxiety]. And it's counterintuitive because you think just calming people down would be a good thing to do. But actually sometimes opening up questions or sort of encouraging people to tolerate the possibility of pain and the possibility of not knowing, yes, that might be a good way to come at it.’

## Man's search for meaning

The tectonic plates of our civilization are shifting uneasily against each other, fault lines exposed. Although there is nothing unique about this pandemic in the course of human history, Grose thinks humans need to tell stories to impose meaning on the chaos. Narratives abound of an anthropomorphized wilderness striking back, to punish us for our destruction of the world.

Grose hates some climate activist's actions that feed into this narrative, ‘[They] were putting up posters saying “human beings are the virus”, and I just thought that was such a nasty way to treat people’. She says, ‘It seems to have been some people in the Midlands trolling XR’ and is upset about how so-called ‘eco-fascists’ use the well-intentioned movement with nefarious intent.

She suggests some alternative narratives:

‘We could say we don't know why it's [the pandemic] happening, but there are ways we could soften the blow. By treating each other well and seeing what we can do and being much more tolerant of each other and prepared to share, less self-interested.’

Extreme messaging may be crude, but it certainly gets people's attention. I ask what is better to inspire behavioural change: appeal to people's desire to protect themselves or to protect others? Grose thinks there are two different types of people, who need different messages. On vegetarianism, she says ‘It's like, you know, meat will give you bowel cancer for one lot. Meat will bring about the apocalypse for the other lot’.

Like many, Grose sees opportunities for our civilization in the ‘Coronapocalypse’, ‘I mean, I just don't see how the world could just slip back to before…Like what is big enough to make people see?’

## ‘An emissary of pity and science and progress, and devil knows what else’

Kurtz, as portrayed by Joseph Conrad, exposes the myth of the Western man's ‘progress’. Grose says that in today's world, we have fooled ourselves into thinking that our technology makes us gods, so that we are estranged from our own mortality. We defend ourselves with material and mind-numbing things. She says:

‘None of these defences are, in themselves, necessarily all bad, but they become a problem when the fantasy solution is opted for so enthusiastically that fashion and cars start clogging up the planet, causing people to feel anxious, and then to vote for yet more of the sorts of leaders who promise an endless supply of fashion and cars.’

She asks that we wean ourselves off ‘these pacifying myths, in order to pave the way to ask proper, difficult questions about life, death and the ethics of coexistence’. She does stress, however, that, ‘It's absolutely vital that environmentalism doesn't equate with miserabilism’.

Grose agrees with the view that eco-anxiety can be a luxury, for those who don't have to worry about their basic survival needs being met. She is clear-eyed about the XR movement being predicated on privilege, in that ‘… being arrested if you're a black person is not the same thing’.

Racism in the discourse around climate change and COVID-19 has been inescapable, Grose agrees. Does our tendency to ‘other’ people, to deny reality until it happens to people like us, betray something about our society? Our tolerance of institutionalized racism shows we have a huge capacity for denial of the ‘other’, Grose thinks:

‘But you're haunted by the fact that your defence isn't working and you know that those people are human. You know that they suffer… And actually, what I hear from people is that the better defended you are in material terms, the more haunted you are.’

Grose looks to Freud to understand why COVID-19 has triggered a retreat into isolationism and jingoism in some countries. The insularity he saw in Germany after World War One represented ‘a state of mourning for what has been lost’, he wrote. She proposes that instead of saying xenophobes and climate change deniers are ‘simply idiots…we could say their minds are somehow in revolt…they don't know what to do about the things they risk losing, or have already lost’.

## Suffer the little children

Babies, says Grose, are ‘the main reason I wanted to write this [eco-anxiety] book’. As a psychotherapist, she has been ‘…dealing for years with people who think it's too risky to have children because the world's going to end’. She has a 19-year-old daughter, so admits that she is biased.

David Attenborough gets some polite digs in her book for his arguments about slowing population growth. It's not poor people in Africa we need to worry about, she says, ‘It's careful, rich people with 1.87 children (or fewer) who constantly upgrade their iPhones…and go on yoga retreats in the Himalayas…An Australian Aboriginal baby has quite a different carbon footprint from, say, a middle-class British one…It's consumerism, not babies, that we need to place the limits on’.

Which brings us to children in general, who are particularly vulnerable to the apocalyptic messaging around us. In her typically frank manner, Grose says there's no easy way to tell children about the climate crisis, just like sex and death:

‘If we're sort of busy pretending it isn't happening and the information's arriving from elsewhere, which it is, then that's not helpful to children…but things that are awful you can only get it wrong. I mean I grew up in a sort of seventies, cool family where sex was completely out there, where, you know there was arty porn around the house. There was the idea that you'd be less traumatized if it was more out in the open. But obviously, it was horrible!’

She thinks we can smooth the conversation by showing it's not completely hopeless but, ‘You can't not get it wrong. Don't be upset if your kids clock you as a worrier – that's just part of being a real parent, rather than one in an advert’.

## Not with a bang but a whimper

It's helpful when overwhelmed, to contemplate your own insignificance, Grose says. ‘When terrestrial life gets too much for you’ she writes, ‘let your mind drift up to the stars. It's so easy to forget they're there’.

I suggest to her that the danger in this is nihilism. Thinking we are unimportant and transient might lead to thinking nothing really matters.

Freud's theory of ‘pre-emptive mourning’ can protect us against this, she says. We are prevented from enjoying the world if we are preoccupied with being ‘fated to extinction’, he observed. Grose offers comfort that ‘…the inevitability of loss and death needn't make us value things any less’. She quotes Freud, ‘A flower that blossoms for a single night does not seem to us on that account less lovely’.

In the rush of COVID-19 think pieces, it's customary to end on a note of optimism, which alleviates the discomfort of our fear. But let's not. Grose reminds us that anxiety is a powerful adaptive force when used productively. ‘The horror, the horror’, Kurtz whispered at the last. Let's sit with that horror and channel it, in the best way that we can.
